# Blocking the tropomyosin receptor kinase A (TrkA) receptor inhibits pain behaviour in two rat models of osteoarthritis

**DOI:** 10.1136/annrheumdis-2014-207203

**Published:** 2015-08-18

**Authors:** Lilian N Nwosu, Paul I Mapp, Victoria Chapman, David A Walsh

**Affiliations:** 1 Arthritis Research UK Pain Centre, University of Nottingham, Nottingham, UK; 2 School of Medicine, University of Nottingham, Nottingham, UK; 3 School of Life Sciences, University of Nottingham, Queen's Medical Centre, Nottingham, UK

**Keywords:** Analgesics, Knee Osteoarthritis, Synovitis, Treatment

## Abstract

**Objectives:**

Tropomyosin receptor kinase A (TrkA) mediates nociceptor sensitisation by nerve growth factor (NGF), but it is unknown whether selective TrkA inhibition will be an effective strategy for treating osteoarthritis (OA) pain. We determined the effects of a TrkA inhibitor (AR786) on pain behaviour, synovitis and joint pathology in two rat OA models.

**Methods:**

Knee OA was induced in rats by intra-articular monosodium-iodoacetate (MIA) injection or meniscal transection (MNX) and compared with saline-injected or sham-operated controls. Pain behaviour was assessed as weight-bearing asymmetry and paw withdrawal threshold to punctate stimulation. Oral doses (30 mg/kg) of AR786 or vehicle were administered twice daily in either preventive (day −1 to –27) or treatment (day 14–28) protocols. Effect maintenance was evaluated for 2 weeks after treatment discontinuation. Alterations in knee structure (cartilage, subchondral bone and synovium) were examined by macroscopic visualisation of articular surfaces and histopathology.

**Results:**

Preventive AR786 treatment inhibited pain behaviour development and therapeutic treatment attenuated established pain behaviour. Weight-bearing asymmetry increased 1 week after treatment discontinuation, but remained less than in vehicle-treated arthritic rats, whereas paw withdrawal thresholds returned to levels of untreated rats within 5 days of treatment discontinuation. AR786 treatment reduced MIA-induced synovitis and did not significantly affect osteochondral pathology in either model.

**Conclusions:**

Blocking NGF activity by inhibiting TrkA reduced pain behaviour in two rat models of OA. Analgesia was observed both using preventive and treatment protocols, and was sustained after treatment discontinuation. Selective inhibitors of TrkA therefore hold potential for OA pain relief.

## Introduction

Osteoarthritis (OA) is a common cause of pain and disability and pain is the most common reason sufferers seek medical help. Despite increased understanding of OA pathogenesis, the mechanisms by which OA is painful remain incompletely understood. Pathological characteristics that have been associated with OA pain include chondropathy, synovitis and subchondral bone marrow lesions.^1–3^ Concurrent with the development of OA, sensitisation of nociceptive pathways augments arthritis pain.^4^ Arthritis pain therefore depends on a combination of pathology within the joint and peripheral and central neuronal sensitisation.

Nerve growth factor (NGF) plays a key role in acute and chronic pain states, especially those associated with inflammation.^5–7^ Sequestering NGF reduces pain in experimental models,^8^ ^9^ and NGF blockade reduces OA pain and improves function in randomised clinical trials^10–12^ more effectively than that observed with non-steroidal anti-inflammatory drugs or opiates.^13^ These data support targeting of NGF pathways for the relief of OA pain. Clinical trials of NGF blockers, although demonstrating analgesic efficacy, also revealed evidence of an increased risk of rapidly progressive OA (RPOA), leading to joint replacement surgery in some treated participants.^14^

Inhibiting the high-affinity NGF receptor TrkA might prevent NGF-mediated sensitisation. Specificity for TrkA might be important in order to avoid possible adverse events from blocking other Trk receptors. For example, disruption of the brain-derived neurotrophic factor(BDNF)-TrkB pathway can lead to hyperphagia and obesity in mice.^15^ Small-molecule, orally active inhibitors might also be more acceptable and cost effective than monoclonal antibodies,^16^ ^17^ but selectivity for TrkA against related tyrosine kinase receptors has proved difficult to achieve. AR786 is a novel orally active inhibitor of TrkA kinase activity. We hypothesise that AR786 might reproduce the analgesic benefits of anti-NGF antibodies.

Reducing sensitisation by NGF signalling blockade is not anticipated to block normal, protective, nociceptive signalling,^18^ unlike traditional analgesics such as opiates. Withdrawal of traditional analgesics that block nociceptive transmission leads to a rapid increase in pain. Peripheral sensitisation is mediated, in part, by altered gene expression,^19^ and inhibitors of sensitisation might be expected to have a slower onset of action but more sustained effect than do directly acting analgesics.^20^

We used AR786 to explore the contribution of the NGF-TrkA pathway to pain behaviour, synovitis and joint pathology in the monosodium-iodoacetate (MIA) and meniscal transection (MNX) rat models of OA. We also investigated the duration of sustained analgesia following withdrawal of treatment.

## Materials and methods

Studies were carried out on male Sprague–Dawley rats (Charles River, Kent, UK), n=100, in accordance with UK Home Office regulations and followed the guidelines of the International Association for the Study of Pain.

### OA induction

Rats weighing 200–250 g were anaesthetised briefly with isoflurane (2% in O_2_) and received a single intra-articular injection of MIA (1 mg/50 µL; Sigma-Aldrich, UK; n=20^21^) in sterile 0.9% saline or underwent transection of the medial meniscus (n=50).^22^ Non-osteoarthritic (saline; n=10 or SHAM; n=20) rats were used as controls. All outcome measurements were carried out by an experimenter blinded to randomised treatments.

### Behavioural measurements of OA pain

Pain behaviour was measured as hindlimb weight-bearing asymmetry and as reduced paw withdrawal thresholds to punctate stimulation of the hind paw.^21^ Baseline measurements were obtained immediately prior to intra-articular injection or surgery (day 0) and every 2–4 days from day 3 onwards to day 21 for the therapeutic study or day 28 for the preventive study. Weight-bearing asymmetry was assessed as percent difference in weight distribution between hindlimbs.^23^

### TrkA inhibitor (AR786) administration

AR786 (Array Biopharma, Boulder, Colorado, USA) was administered in a therapeutic or preventive protocol based on previous data.^17^ ^24^

To evaluate the effects of therapeutic treatment of AR786 in the MIA and MNX models of OA, AR786 (30 mg/kg, orally twice daily) or 5% Gelucire 50/13 vehicle (Gattefosse, Cedex, France) was administered twice daily for 7 days from 2 weeks after OA induction (after OA was established). Rats were stratified into groups of n=10 at day 10 by a researcher not otherwise involved in the study. This was to avoid clinically important differences in weight-bearing asymmetry between arthritic groups that might otherwise occur by chance during randomisation prior to treatment. Rats received either AR786 (MIA or MNX+drug) or vehicle (MIA or MNX+vehicle; saline or SHAM+vehicle).

To evaluate the effects of AR786 preventive treatment and treatment withdrawal in the MNX model, AR786 (30 mg/kg, orally twice daily) or vehicle (5% Gelucire 50/13) was administered 1 h prior to and 8 h following OA induction, and thereafter until the end of the study (day 28 after OA induction). AR786 treatment was discontinued and replaced with vehicle in some rats 2 weeks after continuous treatment. Rats were stratified into treatment groups each comprising 10 animals, 10 days after OA induction (ie, 4 days before treatment discontinuation on day 14). Rats received either AR786 (MNX+drug; MNX+drug-discontinued) or vehicle (MNX+vehicle; SHAM+vehicle).

### TrkA-inhibitor selectivity

Assays were carried out on kinase and non-kinase receptors and channels (see online supplementary text).

### Joint pathology

Rats were killed either by an overdose of CO_2_ (therapeutic study—day 21) or an overdose of pentobarbital (intraperitoneal) (preventive study—day 28). Macroscopic chondropathy scoring was based on the Guingamp classification^25^ (see online supplementary text). Synovium and patellae were removed and frozen in melting isopentane and tibiofemoral joints were preserved in neutral buffered formalin, decalcified and processed.^26^ Histological assessment of cartilage and subchondral bone including osteophytes was based on the Osteoarthritis Research Society International recommendations^27^ (see online supplementary text). Inflammation was assessed as joint swelling as previously described,^26^ and synovitis grade using H&E-stained sections according to lining thickness and cellularity^22^ (see online supplementary text).

### Statistical analysis

Statistical analysis was performed using Prism V.6 (Graph Pad, San Diego, California, USA). At each time point, data were analysed using Kruskal–Wallis test followed by post hoc Dunn's comparison. Area under the curve (AUC) was also used to analyse the pain behaviour data over time. Segments analysed by AUC were determined by treatment changes (eg, before and after commencement of AR786 administration). Graphs are presented as mean±SEM (line graphs) or median (scatter grams). Data in text are presented as mean (95% CI). A two-tailed p<0.05 was considered significant.

## Results

### Selectivity of AR786

AR786 has a greater selectivity for TrkA over a diverse panel of kinases, receptors and ion channels (table 1).

### OA induction by intra-articular injection of MIA or by MNX surgery

**Figure 1:**
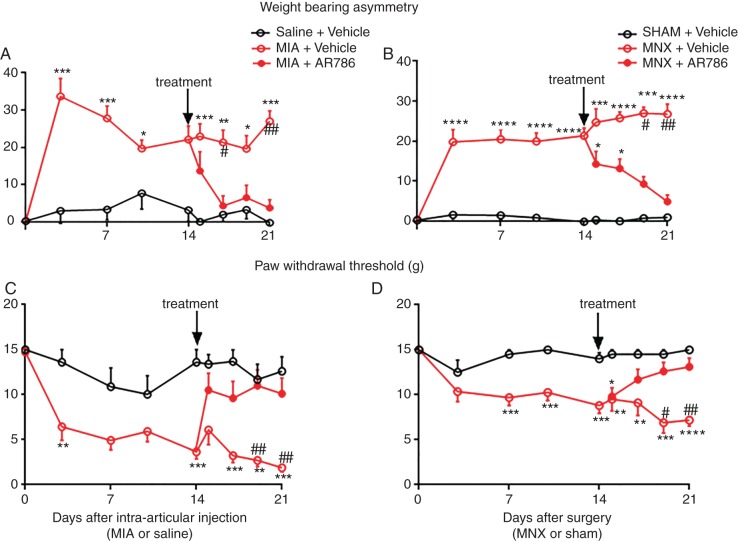
Effect of therapeutic AR786 on pain behaviour in the MIA and MNX models of OA. MIA injection or MNX surgery each was followed by an increase in pain behaviour (weight-bearing asymmetry and paw withdrawal threshold) which was sustained until the end of the study (day 21) (A–D). Administration of AR786 (30 mg/kg twice daily) (treatment) from day 14 completely abolished pain behaviour by day 17 (3 days after start of treatment) in the MIA-injected rats (A and C). In the MNX-operated rats, pain behaviour was attenuated by day 19 (5 days after start of AR786 treatment) and completely abolished to control levels by day 21 (7 days after start of treatment) (B and D). Data indicate mean±SEM for n=10 rats per group. Differences between groups were analysed using Kruskal–Wallis test followed by post hoc Dunn's tests. Significance of post hoc tests is denoted by the number of symbols, for example, *p<0.05; **p<0.01; ***p<0.001. Asterisks (*) denote significant differences from vehicle-treated, saline-injected or sham-operated non-arthritic controls. Hash signs (#) denote significant differences from AR786-treated rats with MIA-induced or MNX-induced OA. MIA, monosodium-iodoacetate; MNX, meniscal transection; OA, osteoarthritis.

**Figure 2:**
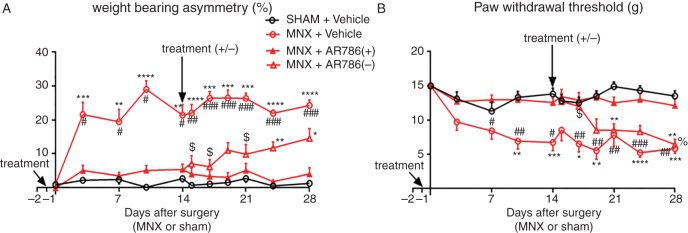
Effect of preventive AR786 treatment on pain behaviour in the MNX model of OA. MNX surgery was carried out on day 0. Either AR786 (30 mg/kg twice daily) (n=20) or vehicle control (5% Gelucire, n=10) was administered orally from 1 day before OA induction (day −1, treatment). AR786 treatment was either continued (n=10), or discontinued and replaced with vehicle treatment (n=10), 14 days after OA induction (treatment ±). MNX surgery was followed in vehicle-treated control rats by increases in pain behaviour (A; weight-bearing asymmetry and B; paw withdrawal threshold) which were maintained until the end of the study (day 28). The development of pain behaviour was completely prevented during the period of AR786 administration. By day 24, 10 days after discontinuation of AR786 treatment in MNX-operated rats, weight-bearing asymmetry was significantly greater than in sham-operated rats, but did not reach the levels observed in MNX-operated, vehicle-treated rats and did not differ significantly from rats that continued to receive AR786 through to the end of the study (day 28) (A). Paw withdrawal thresholds were reduced at day 19, 5 days after treatment discontinuation in MNX-operated rats that had received AR786 treatment until day 14. By this time, paw withdrawal thresholds in MNX-operated rats did not differ significantly between those in which AR786 had been discontinued compared with those that had never received AR786 (MNX-operated, vehicle-treated rats). Paw withdrawal thresholds 7 days after treatment discontinuation in MNX-operated rats were significantly reduced compared with sham-operated, vehicle-treated rats and 14 days after treatment discontinuation paw withdrawal thresholds were reduced compared with MNX-operated rats that continued to receive AR786 (B). Data are mean±SEM of n=10 rats per group. Differences between groups were analysed using Kruskal–Wallis test followed by post hoc Dunn's tests. Significance of post hoc tests is denoted by the number of symbols, for example, *p<0.05; **p<0.01; ***p<0.001. Asterisks (*) denote differences from sham-operated, vehicle-treated control rats. Hash signs (#) denote differences from MNX-operated rats that continued to receive AR786. Dollar signs ($) denote differences in MNX-operated rats after AR786 discontinuation compared with MNX-operated rats that had received vehicle treatment throughout the experiment. Per cent sign (%) denotes differences from MNX-operated rats after AR786 discontinuation compared with MNX-operated rats that continued AR786 treatment. MNX, meniscal transection; OA, osteoarthritis.

Intra-articular injection of MIA, or MNX surgery, was each followed by increased weight-bearing asymmetry from 3 days after arthritis induction, compared with non-arthritic saline-injected or sham-operated controls (figures 1A, B and 2A). Paw withdrawal thresholds ipsilateral to the arthritic knee were reduced by 3 days after arthritis induction in the MIA group and 7 days in the MNX group compared with non-arthritic controls (figures 1C, D and 2B). AUC analysis for both weight-bearing asymmetry and paw withdrawal thresholds confirmed the presence of pain behaviour (see online supplementary figure S1A,C,E,G). Pain behaviour was sustained in arthritic rats through to study termination 21 or 28 days after arthritis induction (figures 1, 2 and see online supplementary figures S1 and S2).

**Table 1:** Trk selective inhibitor AR786 is >1000 fold selective for TrkA over a diverse panel of kinases, receptors, channels and transporters

*Kinases*					*CNS receptors*	*Nociceptive receptors*
AKT1	Flt3	MAPKAP-K2	PKCα	SGK2	Adenosine A2A	Bradykinin B2
AKT2	Flt4	MAPKAP-K3	PKCβI	SGK3	Adenosine A3	Cannabinoid
AKT3	Fms	MAPKAP-K5	PKCβII	SIK	Adrenergic α1	CGRP1
ALK	Fyn	MARK1	PKCδ	SRC	Adrenergic α2	Opiate δ
ALK4	GRK5	MARK2	PKCε	SRPK1	Adrenergic β	Opiate κ
AMPK	GRK6	MEK1	PKCη	SRPK2	Androgen	Opiate µ
ARK5	GRK7	MELK	PKCγ	STK33	Dopamine D1	Phorbolester
AURKA	GSK3α	MKK7β	PKCι	Syk	Dopamine D3	Purinergic
Abl-P	GSK3β	MKNK2	PKCθ	TAK1	GABAA (flunitrazepam)	Tachykinin NK1
Abl2	HIPK1	MLK1	PKCζ	TAO1	GABAA (Muscimol)	Sigma
Ax1	HIPK2	MRCKα	PKD1	TAO2	GABAB	*Inflammatory response*
BLK_m	HIPK3	MRCKβ	PKD2	TAO3	Glutamate	1EP4
BTK	Haspin	MSK1	PRK2	TBK1	GPR103	Glucocorticoid
Bmx	Hck	MSK2	PRKG1α	TLK2	Imidazoline 12	Histamine H1
BrSK1	IGF-1R	MSSK1	PRKG1β	TNK2	Melatonin MT2	Histamine H2
BrSK2	IKKα	MST1	PTK5	TSSK1	Muscarinic (non-selective central)	Histamine H4
CAMK1	IKKβ	MST2	PTK6	TSSK2	Muscarinic oxotremorine	Leukotriene
CAMK1d	IR	MST3	PhKγ2	Tie2	Neuropeptide Y1	PAF
CAMK2b	IR Act	MYLK	Pim-1	Txk	Neurotensin	*CNS transporters*
CAMK2g	IRAK1	Mer	Pim-2	ULK2	Nicotine Acetylcholine	GABA
CAMK4	IRAK4	Met	Pim-3	ULK3	Nicotine Acetylcholine α1	Norepinephrine (NET)
CDK1/cyclinB	IRR	MuSK	Plk1	VRK2	Rolipram	Serotonin (SERT)
CDK2/cyclinA	ITK	NEK11	Plk2	WNK2	Serotonin 5-HT1A	*Channels*
CDK2/cyclinE	JAK2	NEK2	Plk3	WNK3	Serotonin 5-HT2B	Calcium Channel l-Type
CDK3/cyclinE	JAK3	NEK3	PrKX	Yes	Serotonin 5-HT3	Calcium Channel N-type
CDK5/p25	JNK1α1	NEK6	RIPK2	ZAP-70	Somatostatin sst5	*Miscellaneous*
CDK5/p35	JNK2α2	NEK7	ROCK-I	c-Raf	Thyroid Hormone	Endothelin
CDK6/cyclinD3	JNK3	NLK	ROCK-II	eEF-2K		EGF
CDK7/cyclinH/MAT1	KDR	PAK2	Ret	mTOR		VIP1
CDK9/cyclinT1	KIT	PAK3	Ron	mTOR/FKBP12		
CHK1	LIMK1	PAK5	Ros	p38α		
CHK2	LKB1	PAK6	Rse	p38β		
CK1_v	1OK	PASK	Rsk1	p38δ		
CK1δ	Lck	PDGFRα	Rsk2	p38γ		
CK1γ1	Lyn	PDGFRβ	Rsk3	p790S6K		
CK1γ2	MAP3K5	PDK1	Rsk4			
CK1γ3	MAP4K2	PKACα	SGK			

The TrkA selective inhibitor AR786 demonstrated sub-nanomolar cellular inhibition of TrkA (IC50=0.6 nM) and >1000 fold selectivity over TrkB (IC50=1147 nM), and TrkC (IC50=1834 nM). AR786 exhibited a high level of selectivity over a panel of kinases run at the ATP Km at 1.0 and 10 nM compound concentrations respectively as well as a panel of non-kinase receptors and ion channels run at a 10 nM compound concentration. CNS, central nervous system; TrkA, tropomyosin receptor kinase A.

**Figure 3:**
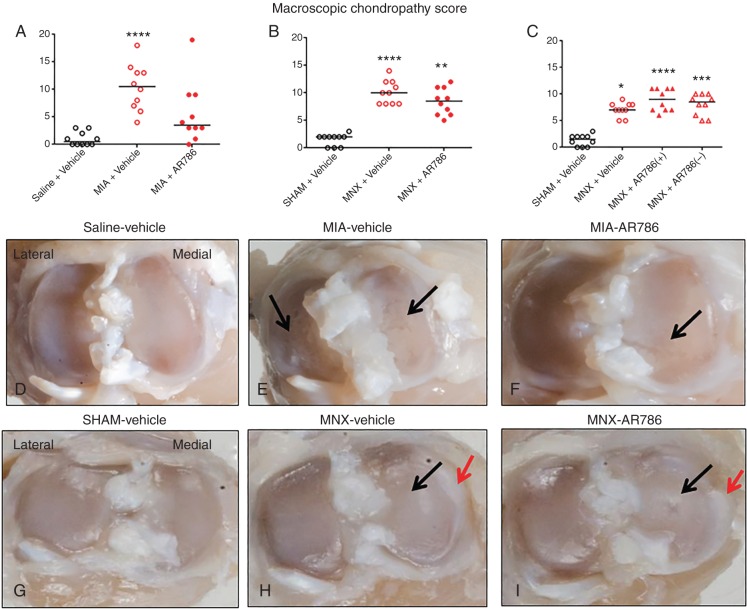
Macroscopic appearances of chondropathy in the MIA and MNX models of OA. Both OA models displayed macroscopic evidence of chondropathy (A–C), 21 days (A and B) or 28 days (C) after arthritis induction. Administration of AR786 had no significant effect on macroscopic evidence of chondropathy in either treatment (A and B) or preventive (C) protocols. Tibial plateaux from 21 days after saline injection (D) or sham operation (G) show normal appearances with no evidence of cartilage erosion. Tibial plateaux from rats 21 days after MIA injection (E and F) or MNX surgery (H and I), and following 7 days treatment with Gelucire vehicle (E and H) or AR786 (F and I) display erosions of the articular cartilage in the weight-bearing areas (black arrows), some of which extend to the underlying subchondral bone. Osteophytosis (red arrows) is also apparent in the medial tibial plateaux of the MNX-operated rats (H and I). Appearances are similar in knees from vehicle-treated and AR786-treated rats. Scoring (A–C) was done using a dissecting microscope at 10 times magnification. Scatter plots show medians of n=10 rats/group. Significance of post hoc tests is denoted by the number of symbols, for example, *p<0.05; **p<0.01; ***p<0.001. Asterisks (*) denote differences from vehicle-treated, saline-injected or sham-operated controls. In (C), MNX-operated, AR786-treated rats either continued to receive AR786 until the end of the experiment (AR786+), or AR786 treatment was discontinued and replaced by vehicle treatment from day 14 until the end of the experiment (AR786−). Photomicrographs (D–I) show medial and lateral tibial plateaux from a rat with the median chondropathy score from each group. MIA, monosodium-iodoacetate; MNX, meniscal transection; OA, osteoarthritis.

Intra-articular MIA injection or MNX surgery was each followed by structural changes to the joint as indicated by increased macroscopic chondropathy scores, cartilage matrix loss, cartilage degeneration score, subchondral bone score and osteophyte score. Macroscopic chondropathy scores after arthritis induction were significantly higher for both vehicle-treated MIA-injected (day 21) (figure 3A) and MNX-operated rats (day 21 and 28) (figure 3B, C) compared with their non-arthritic controls. MIA-induced OA was characterised by macroscopic pathology both in medial and lateral tibiofemoral compartments (figure 3A, E), whereas chondropathy in MNX-induced OA was predominantly localised to the medial tibiofemoral joint (figure 3B, C, H). Cartilage matrix loss, cartilage degeneration scores and subchondral bone scores were significantly higher for the vehicle-treated MIA-injected rats compared with saline-injected rats (see online supplementary figure S3A–C). Cartilage matrix loss, cartilage degeneration scores and osteophyte scores were significantly higher for the vehicle-treated MNX-operated rats compared with the sham-operated rats (see online supplementary figure S3E, F, H).

**Figure 4:**
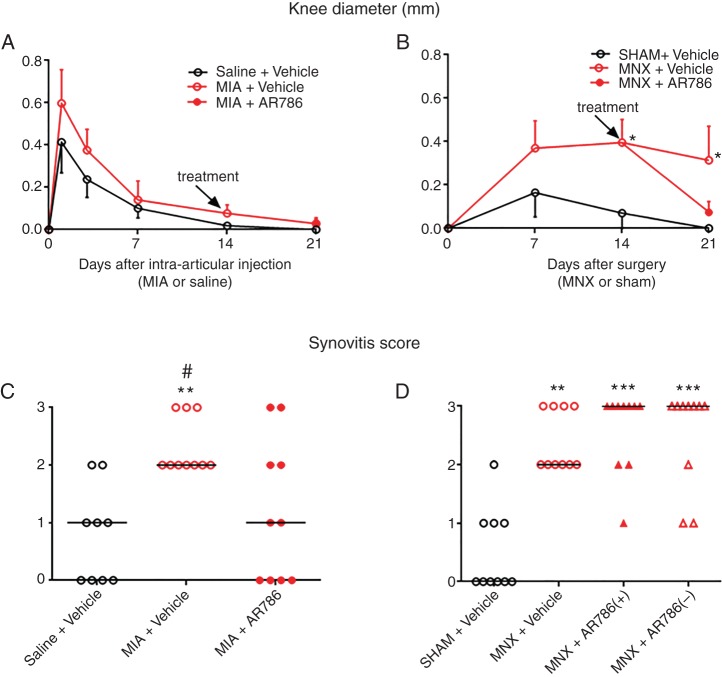
Effect of AR786 on MIA-induced or MNX-induced knee inflammation. Small changes in knee diameter were observed after intra-articular injection of MIA or saline (A), or after MNX surgery or sham surgery (B), as indicated by small differences between injected/operated and contralateral knees. Either AR786 (30 mg/kg twice daily) or vehicle (5% Gelucire) control was administered orally from day 14 (treatment). Knee diameters did not differ significantly between MIA-injected or saline-injected knees, nor between MIA-injected rats treated with either AR786 or vehicle (A). Knee diameter differences between operated and contralateral, non-operated knees were greater in MNX-operated than in sham-operated rats from 14 days after surgery (B). Differences in knee diameter between AR786-treated and vehicle-treated, MNX-operated rats at day 21 (7 days after the start of treatment) did not reach statistical significance. Synovitis scores, determined by histology, were significantly higher for MIA-injected rats (C) at 21 days and MNX-operated rats (D) at 28 days compared with saline-injected or sham-operated controls. Synovitis scores were reduced in MIA-injected rats following treatment for 7 days with oral AR786 compared with Gelucire vehicle-treated controls (C). Synovitis scores were not inhibited by preventive administration of AR786 in MNX-operated rats (D). Data are median of n=10 rats/group (A and B). Significance of post hoc tests is denoted by the number of symbols, for example, *p<0.05; **p<0.01; ***p<0.001. Asterisks (*) denote differences from saline-injected or sham-operated, vehicle-treated control rats. Hash sign (#) denotes differences from MIA-injected rats that received AR786. In (D), MNX-operated, AR786-treated rats either continued to receive AR786 until the end of the experiment at day 28 (AR786+), or AR786 treatment was discontinued and replaced by vehicle treatment from day 14 (AR786−). No significant differences were observed between continued and discontinued treatment groups. MIA, monosodium-iodoacetate; MNX, meniscal transection.

**Figure 5:**
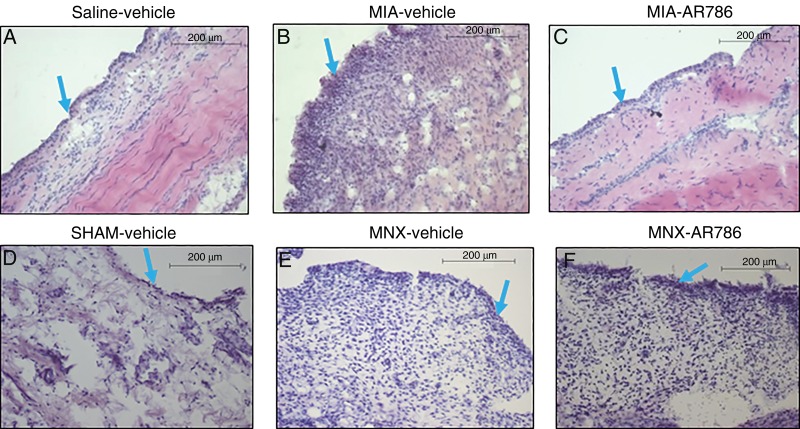
Effect of AR786 on MIA-induced or MNX-induced synovitis. Synovia collected 21 days after saline injection and 7 days oral treatment with Gelucire vehicle (A) or 28 days after sham operation and 28 days oral treatment with Gelucire vehicle (D) display normal microscopic appearances. Both OA models displayed microscopic evidence of synovitis. MIA injection (B and C), or MNX operation (E and F) resulted in mild synovitis, characterised by increased thickness of the lining layer (blue arrows), and increased sublining cellularity. Synovitis was reduced in MIA-injected rat knees following treatment for 7 days with oral AR786 (C) compared with Gelucire vehicle-treated controls (B). Synovitis after MNX surgery appeared similar in rats treated either with AR786 (F) or vehicle (E). Photomicrographs show H&E-stained sections of synovium from a rat with the median synovitis score from each group. Scale bars represent 200 μm. MIA, monosodium-iodoacetate; MNX, meniscal transection; OA, osteoarthritis.

Small increases in knee diameter were observed from 3 days following intra-articular injection of MIA, but did not significantly differ from saline-injected rats (figure 4A). MNX or sham surgery each resulted in increased knee diameter that persisted in MNX-operated rats, remaining significantly greater than sham-operated controls from 14 days after surgery (figure 4B). Synovitis scores detected by histological examination were significantly increased after MIA injection (day 21) (figures 4C and 5A, B) or MNX surgery (day 28) (figures 4D and 5D, E) compared with their non-arthritic controls.

### Effects of TrkA inhibitor on established OA pain behaviour

Rats received AR786 for 7 days from 2 weeks after OA induction (at a time shown previously to correspond to fully established OA joint pathology and pain behaviour both in MIA-induced and MNX-induced models).^28^ Weight-bearing asymmetry was reduced following 3 days oral administration of AR786 in rats with MIA-induced OA (4.3 (−1.9 to 10) %) and following 5 days treatment in MNX-induced OA (9.2 (5.1 to 13) %)) compared with vehicle-treated arthritic rats at the same time points (MIA (21 (14 to 29) % p=0.02, figure 1A) and MNX (27 (24 to 30) % p=0.02, figure 1B)). These reductions in weight-bearing asymmetry following oral administration of AR786 were confirmed using AUC analysis (see online supplementary figure S1B,D). Paw withdrawal thresholds were also increased following 5 days treatment with AR786 in rats with MIA-induced OA (11 (7 to 15) g) or MNX-induced OA (13 (10 to 15) g)) compared with vehicle-treated arthritic rats (MIA (2.7 (1.2 to 4.2) g p=0.004 figure 1C) and MNX (6.9 (4.3 to 9.5) g p=0.01), figure 1D). Increases in paw withdrawal thresholds following AR786 administration were confirmed in MIA-induced OA using AUC analysis, whereas differences in MNX-induced OA did not reach statistical significance (see online supplementary figure S1). Both pain behaviours in arthritic rats after treatment with AR786 were comparable with those in non-arthritic controls, irrespective of the mode of arthritis induction (figure 1).

### Effects of TrkA inhibition on the development of OA pain behaviour

Rats received AR786 1 day prior to, during and for 28 days after OA induction by MNX. Treatment with AR786 was withdrawn in one group of rats (n=10) 2 weeks after arthritis induction and replaced with vehicle treatment. Weight-bearing asymmetry was prevented in MNX-operated rats at all time points when they received AR786 throughout the study (day 28; (4 (−0.1 to 8.1) %) compared with vehicle-treated, MNX-operated rats (24 (21 to 28) %, p<0.001, figure 2A)). Likewise, paw withdrawal thresholds were comparable with non-arthritic control values in arthritic rats that received AR786 throughout the study (day 28; (12 (9.9 to 14) g) and significantly different from values in vehicle-treated MNX-operated rats (5.8 (4.2 to 7.4) g, p=0.003, figure 2B). AUC analysis for both weight-bearing asymmetry and paw withdrawal threshold showed prevention of pain behaviour with continued AR786 administration (see online supplementary figure S2A–D).

Withdrawal of AR786 treatment was followed by continuing reduced weight-bearing asymmetry for 10 days compared with arthritic rats that had never received the drug (figure 2A). Arthritic rats that had their treatment withdrawn did not show a statistical significant difference in weight-bearing asymmetry compared with arthritic rats that had continued treatment through to the end of the study (figure 2A). By contrast, paw withdrawal thresholds rapidly decreased following withdrawal of AR786, and 5 days after treatment withdrawal were comparable with paw withdrawal thresholds in rats with MNX-induced OA that never received AR786 (figure 2B). Paw withdrawal thresholds in the arthritic rats that had their treatment withdrawn were significantly lower than in arthritic rats with continued treatment at the end of the study (day 28) (figure 2B).

### Effects of TrkA inhibition on OA joint pathology

We investigated whether observed effects of TrkA inhibition on pain behaviour might be mediated or moderated by OA joint pathology.

AR786 had no significant effect on joint swelling (figure 4A, B) or on macroscopic chondropathy in either OA model, whether administered in treatment or preventive protocols (figure 3). AR786 had no significant effect on cartilage surface integrity, subchondral bone or on osteophyte formation in either OA model (see online supplementary figure S3). Administration of AR786 for 7 days commencing from 14 days after OA induction by intra-articular injection of MIA was associated with significant reductions in synovitis scores compared with vehicle-treated, MIA-injected rats (figure 4C). However, no significant reductions in synovitis scores were observed at 4 weeks following continuous preventive administration of AR786 in rats with MNX-induced OA (figure 4D).

## Discussion

We have investigated effects of a novel selective TrkA inhibitor, AR786 in two rat OA models. OA-associated pain behaviour was inhibited by AR786 using both preventive and therapeutic protocols. AR786 also reduced synovitis in one of the models, whereas we detected no significant effect on osteochondral pathology. Our data suggest potential of a small-molecule, orally available, selective TrkA inhibitor for the treatment of OA pain.

Intra-articular injection of MIA, or MNX surgery, led to OA pathology (chondropathy, subchondral bone pathology, osteophytes and mild synovitis) and increased pain behaviours, as previously reported.^26^ ^29^ Weight-bearing asymmetry, comparable with standing pain in people with OA,^30^ might result from a combination of nociception and sensitisation.^31^ Reductions in hind paw withdrawal thresholds to punctate mechanical stimulation are associated with central sensitisation in rats with OA.^21^ Central sensitisation might augment joint pain and contribute to more widespread pain sensitivity in people with OA.^31^ Sequential progression from peripheral to central nociceptive mechanisms during the induction of OA in rats is suggested by early weight-bearing asymmetry followed by reduced paw withdrawal thresholds, and is further indicated by electrophysiological, cellular and molecular studies in rat OA models.^32^ ^33^

Minor differences in pain phenotypes reported between MIA and MNX OA models might reflect heterogeneity also observed between different people with OA. Reduced paw withdrawal thresholds in MIA-induced OA were more pronounced than in MNX-induced OA, as previously reported,^28^ consistent with a greater contribution from central pain processing.^34^ ^35^ Consistent analgesic effects of TrkA inhibitors between models suggest that our findings might be relevant to OA in general, rather than being specific to the mode of OA induction. However, caution must be exercised before generalising from laboratory findings to human OA. Rat models develop rapidly and pain mechanisms might differ in early compared with long established OA. Although rat OA models display similar histological and pain behavioural characteristics to those observed in chronic human OA,^29^ preclinical studies can only guide, rather than accurately predict, results of research in humans.

Oral AR786 administration abolished pain behaviour in both MIA and MNX models in both preventive and therapeutic studies. Our findings extend previous reports of analgesic effects of Trk inhibitors in non-malignant skeletal and bone cancer pain^17^ ^24^ and indicate that analgesic effects of Trk inhibition might be mediated specifically by TrkA. NGF blockade using a TrkA–IgG fusion protein also reduced pain behaviour in rodent OA.^9^ Our findings indicate a role also for the high-affinity NGF receptor, TrkA, and demonstrate the potential for a small-molecule selective inhibitor of TrkA as a novel therapeutic strategy in OA.

AR786 displays low central nervous system penetration, with plasma:CSF (cerebrospinal fluid) ratios approximately 10:1.^36^ However, TrkA inhibition attenuated reduced paw withdrawal thresholds, a behavioural correlate of central sensitisation. NGF might induce central sensitisation indirectly by increasing BDNF release from primary afferent neurons.^37^ ^38^ Furthermore, synovitis might drive central sensitisation during arthritis.^26^ Increased paw withdrawal thresholds relapsed more quickly after treatment withdrawal than did weight-bearing asymmetry, suggesting that TrkA inhibition has a more sustained effect on peripheral pain processing than on central sensitisation. It is likely that increases in paw withdrawal thresholds following TrkA inhibition result from blocking peripheral actions of NGF, although further research would be required to determine whether low concentrations of AR786 in the CSF could alter central pain processing.

We demonstrate that rats with OA that received 14 days pre-emptive treatment continued to display improved weight-bearing asymmetry for at least 10 days after treatment withdrawal, despite displaying macroscopic evidence of OA pathology. This raises the potential that TrkA inhibition might prevent the transition to a sensitised state if administered for short periods during critical phases of OA development. This contrasts, for example, with drugs such as indomethacin, which, while reducing weight-bearing asymmetry in MNX-induced OA, require continued treatment for maintenance of effect, with a relapse of weight-bearing asymmetry to levels that are comparable with those seen in arthritic rats that have never received indomethacin only 3 days after treatment withdrawal.^26^ ^32^

Synovitis is associated with pain in human OA.^2^ Inflammation generates factors that activate or sensitise joint nociceptors, including NGF.^39^ TrkA activation increases inflammatory and sensitising neuropeptide release from sensory nerves, including substance P and calcitonin gene-related peptide.^40–42^ Furthermore, TrkA might be expressed by mast cells,^43^ synovial fibroblasts^44^ and macrophages^45^ and inflammatory cell activation by NGF leads to the upregulation and release of inflammatory mediators, which might further activate or sensitise joint nociceptors.^46^ ^47^ AR786 reduced synovitis in rats with MIA-induced OA, but did not significantly reduce either knee swelling or synovitis in rats with MNX-induced OA. These apparent differences might reflect different mechanisms or severity^28^ of inflammation between the two models. Further research would be required to determine the mechanisms underlying the anti-inflammatory effects of AR786, and whether human OA displays AR786-sensitive inflammation.

Chondropathy is a hallmark of human OA,^48^ and cartilage changes are associated with pain severity.^49^ Clinical trials of NGF-blocking antibodies were put on hold due to the rare occurrence of RPOA.^50^ Small reductions in macroscopic and microscopic chondropathy scores observed following treatment with AR786 did not reach statistical significance and are unlikely to have made any major contribution to the observed reductions in pain behaviour. However, more sustained synovitis inhibition might facilitate pain relief in OA in part by reducing joint damage.^51^ Our studies were not powered to detect rare adverse effects on joint structure.

In conclusion, the inhibition of NGF activity by blocking TrkA reduced pain behaviour in two rat models of OA. Reductions in pain behaviour were observed both using preventive and treatment protocols, and were sustained for up to 10 days despite treatment discontinuation. Clinical trials of NGF blockade have demonstrated potential of blocking the NGF/TrkA pathway for the relief of OA pain. Further research should address possible differences between NGF-blockade and selective TrkA inhibition, and the potential for sustained benefit from discontinuous treatment, in order to help realise the potential of NGF pathway inhibition for OA pain relief.

## Supplementary Material

Web supplement
