# Supplementary material for: Blocking the tropomyosin receptor kinase A (TrkA) receptor inhibits pain behaviour in two rat models of osteoarthritis
Source: Ann Rheum Dis. 2015 Aug 18;75(6):1246–54. doi: 10.1136/annrheumdis-2014-207203 (PMC4893148; doi:10.1136/annrheumdis-2014-207203)

Methods

Trk-inhibitor selectivity

Kinases. Kinase Profiler assay (Millipore, MA, USA) consisting of 229 radiometric protein kinase assays was used. Each specific kinase was incubated with a 3-(N-morpholino) propanesulfonic acid (MOPS) buffer, peptide specific to the kinase assay, magnesium acetate and gamma^{33}P-ATP. The reaction was initiated by the addition of the magnesium ATP mix. After incubation at room temperature the reaction was stopped using a phosphoric acid solution. An aliquot of the reaction was then spotted on a filter, washed with phosphoric acid, then methanol once prior to drying and scintillation counting. Percent of Control (POC) data were determined for AR786 at compound concentrations of 1 µM and 10 µM respectively.

Non-kinase receptors and channels. Assays were from Ricerca Biosciences, Concord, Ohio. Each assay was run in duplicate at 10 µM. The radioligand binding assay source was human recombinant cells or mouse, Guinea pig or rat tissue specific for each assay. The radiolabeled ligand was specific for each receptor and incubations were performed in a neutral pH buffer with compound or a non-specific ligand for a control. Incubation time and conditions vary for each assay with significance being defined as >50% stimulation or inhibition of ligand binding.

Joint pathology

Macroscopic chondropathy scoring of knee joints was based on the Guingamp classification,[1], on a scale from Grade 0 = normal appearance, 1 = slight yellowish discolouration of the chondral surface, 2 = little cartilage erosion in load bearing areas, 3 = large erosions extending down to the subchondral bone and 4 = large erosions with large areas of subchondral bone exposure. Five chondral compartments of the knee: femoral groove, medial and lateral femoral condyles and medial and lateral tibia plateaux were scored. The 5 compartment scores were summated to give a maximum possible score of 20.

Histological assessments

Frontal sections following the Osteoarthritis Research Society International (OARSI) guideline for histological assessment for OA in the rat,[2] were cut and stained with H&E. Three sections per rat at 200µm intervals were stained and scored for cartilage matrix loss, cartilage degeneration, calcified cartilage and subchondral bone damage and osteophytes in the MNX model, whereas 6 sections per rat were used for the MIA model.

Cartilage matrix loss was scored based on the depth of cartilage degeneration. 0% = cartilage loss on the surface, 50% = cartilage loss down to midzone and 100% = cartilage loss at the level of the tidemark.

Cartilage degeneration was scored from 0 = no degeneration, 1 = minimal degeneration (5-10% affected), 2 = mild degeneration (11-25% affected), 3 = moderate degeneration (26-50% affected), 4 = marked degeneration (51-75% affected) to 5 = degeneration (greater than 75% affected). Each score was multiplied by the number of thirds of the cartilage length that was involved (1, 2 or 3) to give a maximum possible score of 15.

Calcified cartilage and subcondral bone damage were scored from 0 = no changes to 5 = collapsed articular cartilage into the epiphysis to a depth of greater than 250µm from tidemark.

Osteophytes were scored 0 = marginal zone proliferative changes <200µm, 1 = small 200-299µm, 2 = moderate 300-399µm, 3 = large 400-499µm or 4 = very large ≥500µm.

Synovitis

Synovitis scoring was performed using a 20× objective lens of a Zeiss Axioscop-50 microscope (Carl Zeiss Ltd, Welwyn Garden City, UK). Synovitis grade was assessed on haematoxylin and eosin-stained sections according to lining thickness and cellularity on a scale from 0 = Lining cell layer 1–2 cells thick, 1 = 3-5 cells thick, 2 = 6-8 cells thick and/or mild increase in cellularity to 3 = lining cell layer >9 cells thick and/or severe increase in cellularity,[3].

References

1. Guingamp C, Gegout-Pottie P, Philippe L, et al. Mono-iodoacetate-induced experimental osteoarthritis: a dose-response study of loss of mobility, morphology, and biochemistry. Arthritis Rheum 1997;**40**(9):1670-9.
2. Gerwin N, Bendele AM, Glasson S, et al. The OARSI histopathology initiative - recommendations for histological assessments of osteoarthritis in the rat. Osteoarthritis Cartilage 2010;**18 Suppl 3**:S24-34.
3. Mapp PI, Avery PS, McWilliams DF, et al. Angiogenesis in two animal models of osteoarthritis. Osteoarthritis Cartilage 2008;**16**(1):61-9.

Supplementary Figures

Supplementary Figure 1. Effect of therapeutic AR786 on pain behaviour in the MIA and MNX models of OA

Area under the curve (AUC) data show increased weight bearing asymmetry (A and C) and reduced paw withdrawal thresholds (E and G) in MIA-injected or MNX-operated rats compared with saline-injected or sham-operated control rats, during the period in which rats were either untreated, or received control vehicle (5% Gelucire), up to 21 days after model induction. AR786-treatment significantly attenuated weight bearing asymmetry in both OA models (B and D), compared to vehicle-treatment, whereas reductions in AUC for paw withdrawal thresholds only reached statistical significance in the MIA-induced model (F). Symbols show data for individual rats and median. Asterisks (*) denote differences from saline-injected or sham-operated, vehicle-treated control rats. Hash signs (#) denote differences from MIA-injected or MNX-operated rats that received AR786. Significance of post hoc tests is denoted by the number of symbols, eg: *: p<0.05; **: p< 0.01; ***: p<0.001. Data are from the same experiment as shown in Figure 1.

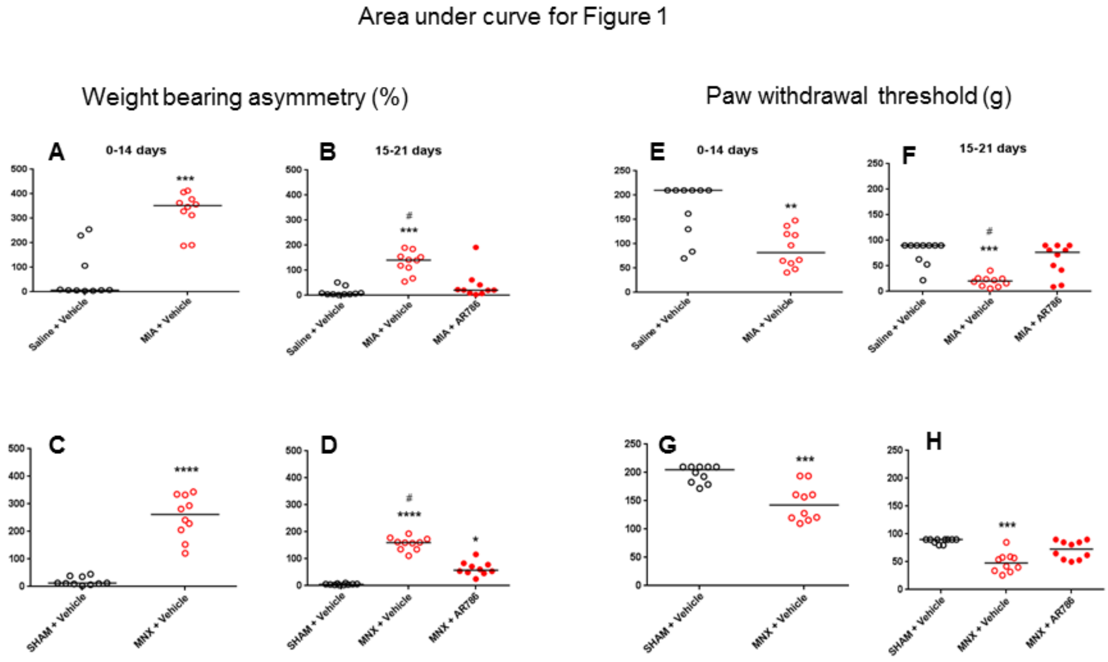

Supplementary figure 2: Effect of preventive AR786 on pain behaviour in the MNX model of OA

Area under the curve (AUC) data show increased weight bearing asymmetry (A, B) and reduced paw withdrawal threshold (C, D) for MNX-operated, vehicle-treated rats over 4 weeks compared to the sham-operated, vehicle-treated controls. Continuous preventive treatment with AR786 significantly both inhibited weight bearing asymmetry and increased paw withdrawal thresholds in MNX-operated rats over 4 weeks. Discontinuation of AR786 (MNX+AR786-) led, over the following 2 weeks, to an attenuation of the reductions in weightbearing asymmetry (B) and increases in paw withdrawal thresholds (D) compared to rats that continued to receive AR786 (MNX+AR786+). Data points represent individual rats and median. Significance of post hoc tests is denoted by the number of symbols, eg: *: p<0.05; **: p< 0.01; ***: p<0.001. Asterisks (*) denote differences from sham-operated vehicle-treated controls. Hash signs (#) denote differences from MNX-operated rats that continued to receive AR786. Data are from the same experiment as shown in Figure 2.

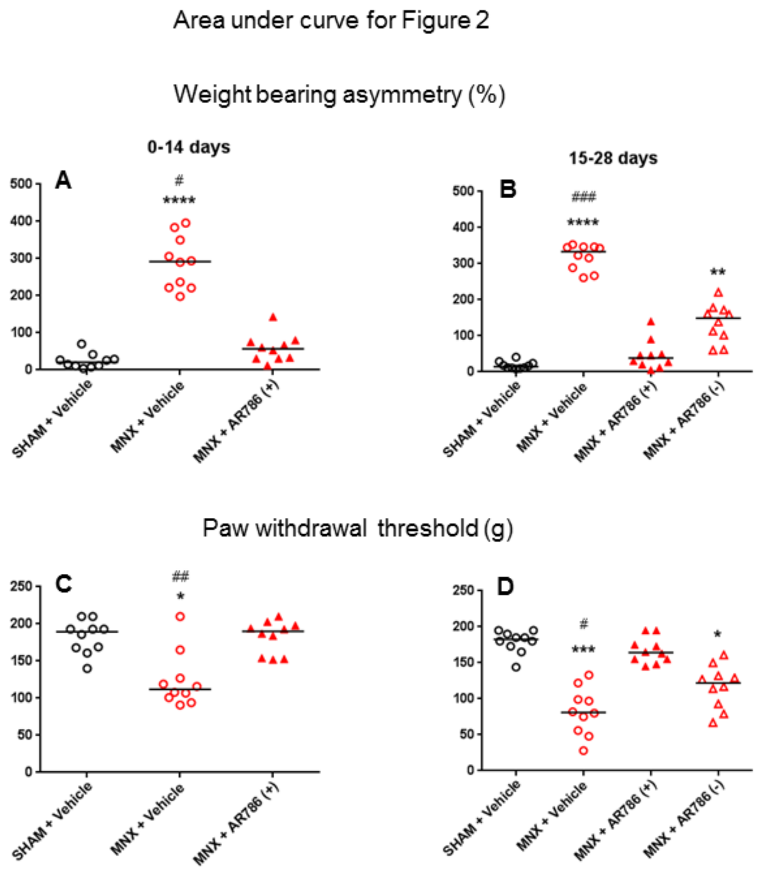

Supplementary figure 3: Cartilage and bone pathology in the MIA and MNX models following AR786 or vehicle treatment

Both OA models 21 days after induction displayed greater microscopic evidence of cartilage matrix loss (A, D) and cartilage degeneration (B, E) than did saline-injected or sham-operated rats. Scores for OA pathology in the calcified cartilage and subchondral bone (C, F) were significantly higher in MIA-injected than in saline-injected rats, and osteophyte scores (D, G) were significantly higher in MNX-operated than in sham-operated rats. AR786-treatment from day 14 had no significant effect on cartilage and bone pathology scores compared to vehicle-treated controls. Data points represent individual rats and median. Asterisks (*) denote differences from saline-injected or sham-operated controls (*: $p<0.05$; **: $p< 0.01$; ***: $p<0.001$).

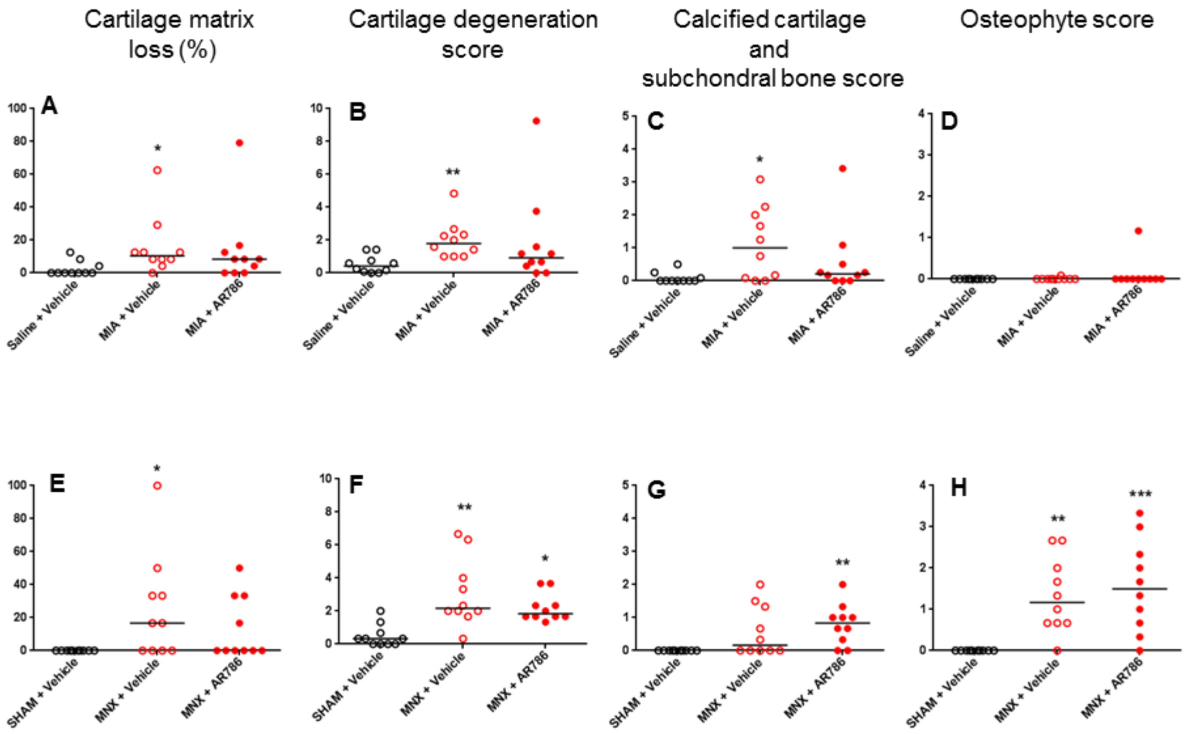

Supplement: Web supplement [file annrheumdis-2014-207203-s1.pdf]
